# Intakes of whole grain in an Italian sample of children, adolescents and adults

**DOI:** 10.1007/s00394-015-1097-5

**Published:** 2015-11-20

**Authors:** Stefania Sette, Laura D’Addezio, Raffaela Piccinelli, Sinead Hopkins, Cinzia Le Donne, Marika Ferrari, Lorenza Mistura, Aida Turrini

**Affiliations:** 1 CREA - Consiglio per la ricerca in agricoltura e l’analisi dell’economia agraria – Centro di ricerca Alimenti e Nutrizione, Via Ardeatina 546, 00178 Rome, Italy; 2 Cereal Partners Worldwide, Lausanne, Switzerland

**Keywords:** Whole grain, Nutrients, Food source, Diet quality

## Abstract

**Purpose:**

There is wide evidence that regular consumption of whole grain foods may reduce the risk of chronic diseases. The aim of this work was to quantify the intake of whole grains and identify main dietary sources in the Italian population.

**Methods:**

Whole grain intakes were calculated in a sample of 2830 adults/older adults and of 440 children/adolescents from the last national survey INRAN-SCAI 2005–06. Food consumption was assessed from a 3-day food record. The whole grain content of foods was estimated mainly from quantitative ingredient declarations on labels.

**Results:**

Mean whole grain intakes were 3.7 g/day in adults/older adults and 2.1 g/day in children/adolescents. Overall, 23 % of the sample reported consumption of whole grain foods during the survey, among which mean whole grain intakes ranged from 6.0 g/day in female children to 19.1 g/day in female older adults. The main sources of whole grains were breakfast cereals in children/adolescents (32 %) and bread in adults/older adults (46 %). Consumption of whole grain among adults was associated with significantly higher daily intakes and adequacy of dietary fibre, several vitamins (thiamine, riboflavin, vitamin B_6_) and minerals (iron, calcium, potassium, phosphorus, zinc, magnesium) compared to non-consumption. Among children, whole grain intake was associated with significantly higher intakes of iron and magnesium.

**Conclusions:**

The study reveals very low whole grain intakes across all age groups of the Italian population. Considering the positive association in consumers between whole grain intakes and fibre and micro-nutrient intakes, public health strategies to increase whole grain consumption should be considered.

**Electronic supplementary material:**

The online version of this article (doi:10.1007/s00394-015-1097-5) contains supplementary material, which is available to authorized users.

## Introduction

Numerous epidemiological studies provide evidence that consumption of whole grains as part of a balanced diet may reduce the risk of chronic diseases such as cardiovascular disease (CVD), type 2 diabetes and some types of cancer (mainly gastrointestinal); moreover, a habitual consumption of whole grain foods may contribute to weight management [1–7]. Findings from randomized controlled trials have been less consistent with some studies showing positive effects of a diet rich in whole grain foods on blood pressure [8], insulin sensitivity [9] and plasma cholesterol [10] and others showing no effects on these outcomes [11–14]. The mechanism of action of these beneficial effects is not clear, but it is likely due to the synergy of many bioactive components present in whole grain products, i.e. dietary fibre, vitamin E, a range of B vitamins, minerals and phytochemicals that may have a protective role with regard to health [15]. Indeed, a moderate consumption of whole grains (usually one to three servings per day equating to 16–48 g/day) has been associated with a more adequate nutrient intake and better diet quality in several populations [16–21].

Although there is no globally accepted definition for whole grain, the definition proposed by the American Association of Cereal Chemists International (AACCI) has been widely adopted and states that “whole grains consist of the intact, ground, cracked or flaked caryopsis whose principal anatomical components—the starchy endosperm, germ and bran—are present in the same relative proportions as they exist in the intact kernel” [22]. Recently, the consortium of the HEALTHGRAIN EU project [23] published a more comprehensive definition with the aim of harmonizing current EU definitions and to better reflect industry practices for the production of flour and consumer products. While similar to the AACCI definition, the HEALTHGRAIN definition allows for small losses of the total grain (<2 %) and bran (<10 %)—that occur through processing methods consistent with safety and quality. With regard to what constitutes a whole grain food, there is a general lack of consensus currently among scientists and national regulatory bodies.

The average intake of whole grain in both adults and children remains very low in America and Europe ranging from 4 to 55 g/day in children/adolescents and from 5 to 55 g/day in adults [17–21, 24–28]. In the UK, 18 % of adults (19+ years) and 15 % of children (1.5–18 years) do not consume any whole grain and only around one-fifth of adults and children consume one serving/day (equal to 16 g/day) [20]. Similarly, in the USA only 3 % of children/adolescents (2–18 years of age) and 8 % of adults (≥19 years of age) consume at least three 16 g servings per day [29]. In contrast, whole grain intakes tend to be higher in Scandinavian countries. In Denmark, whole grain intakes increased from 28 to 54 g/day in children (4–14 years old) and from 32 to 55 g/day in adults (15–75 years old) following the successful implementation of the Danish Whole Grain Campaign [24, 28].

In 2014, the WHO and EU published the revised European Code against Cancer, in which it is recommended to have a healthy diet with inclusion of plenty of whole grains, pulses, vegetables and fruits [30]. Moreover, the consumption of whole grains is recommended in the dietary guidelines of many countries. These guidelines range from being quantitative in the USA (48 g/day) and Denmark (75 g/10 MJ), to non-specific advice such as “products made from grains such as bread, pasta, rice should be preferably whole grain” in many other countries including UK [31] Germany [32] Greece [33], France [34] and also Italy [35]. The recommendation presented in the most recent Mediterranean Diet pyramid [36] provides more quantitative guidance on the consumption of whole grain cereals, recommending the consumption of one or two servings of cereals at each main meal (in the form of bread, pasta, rice, couscous and others) and preferably whole grains.

In European countries, particularly in southern Europe, there is a lack of information on whole grain intakes. The aim of the present study was to perform a secondary analysis of the Italian food consumption database INRAN-SCAI 2005–06 to estimate whole grain intakes and major food sources of whole grains in children/adolescents and adults/older adults and to examine the association of whole grain consumption with daily nutrient intakes and adequacy.

## Methods

### Study population and data collection

The INRAN-SCAI 2005–06 study was a cross-sectional survey conducted on a representative sample of 1300 households randomly selected and stratified into the four main geographical areas of Italy (North-West, North-East, Centre, South and Islands) between October 2005 and December 2006. In total, 1329 households participated in the food survey corresponding to 3323 individuals (1501 males and 1822 females), aged 0.1–97.7 years. Detailed information about the INRAN-SCAI 2005–06 survey design, procedures and methodologies can be found on the previous published papers [37, 38].

A 3-day semi-structured diary was used to collect the food consumption of each subject. Participants recorded all foods and drinks consumed both inside and outside the home over 3 consecutive days. The quantity consumed for each food/beverage/supplements was determined using household measures and estimated portion sizes according to detailed guidance notes (with instructions to quantify the portions used by children) and photographs atlas developed on the basis of EPIC-SOFT picture book. For children below 8 years and for any subject who was not able to do so, the diaries were filled in by the person who took care of him/her. Moreover, information on the brand of manufactured and packaged foods was collected as much as possible, mainly for fortified foods and supplements.

For each participant, self-reported height and weight were recorded. Information on socio-demographics (education, occupation, marital status), lifestyle (smoking, dieting, dietary pattern—Mediterranean/traditional vs others: vegetarian, vegan, fruitarian, macrobiotic, etc.—physical activity, sedentary activity, use of supplements and fortified foods, out-of-home meals) and nutritional knowledge variables (knowledge of diet–health relationship, frequency of reading food labels) was determined by a self-administered questionnaire at the time dietary records were collected. For children/adolescents, the information available was limited to the level of education of the family (highest level among adult family members), physical activity and hours of sedentary activity per day. In order to capture all seasonal differences in intake, the sampled households were proportionally distributed among seasons (excluding Christmas and Easter periods): 25 % in autumn, 25 % in winter, 26 % in spring and 24 % in summer. In addition, the survey calendar was scheduled to take an adequate proportion of weekdays and weekend days at group level (78 and 22 %).

The survey was purely observational and non-invasive; ethical aspects were related only to the collection of information on food habits that may be related to health and thus might be sensitive. At the time of the survey, INRAN institute was part of the National Statistical System (SISTAN) and adhered to the principle of statistical confidentiality, moreover, as Public Body INRAN adopted the current regulation on guarantees individual data protection. An additional ethical committee review of the study protocol was considered unnecessary.

For the present study, all individuals above 3 years of age were considered; only one female subject (aged 66 years) that declared to be on a specific diet (high consumption of bran and whole grain products) during the survey was excluded. The sample was subdivided into two age groups: children/adolescents (3- to 17-year-old individuals, no. 440) and adults/older adults (individuals of age 18 and above, no. 2830). Data on energy and nutrients intake were obtained using the updated version of national food composition database [38]. In the case of foods and beverages that were fortified or enriched with one or more essential nutrients (included functional foods and foods for special purpose), the nutrient content was retrieved at brand level from nutritional labels.

### Calculation of whole grain intakes

For the purpose of this study, the term “whole grain” was defined in accordance with that outlined by HEALTHGRAIN [23], as presented in the introduction of this paper. It should be noted, however, that Italian regulation permits the use of the term “whole grain” to be applied to products made from whole wheat flour purchased as such from milling companies and also to products made with white flour to which varying amounts of bran have been added back. In the first case, “whole wheat flour” is listed as one ingredient, but in the latter case, the ingredients are listed separately (wheat flour, bran, middling) [39]. For the present analysis, products containing “whole wheat flour” and products made with oats, rice, maize/corn, barley, rye and other cereals were considered.

Of the fifty-one original food sub-categories in the INRAN-SCAI food consumption database, nine were identified as containing whole grain (“Bread”, “Pasta and pasta substitutes”, “Rice”, “Wheat, other cereals and flours”, “Breakfast cereals”, “Biscuits”, “Savoury fine bakery products”, “Cakes and sweet snacks”). In addition, the food groups “Yoghurt and fermented milk”, “Milk based desserts and substitutes”, “Ice cream, ice lolly and substitutes” and “Miscellaneous” were also checked for the presence of whole grain ingredients, as in the case of yogurt with cereals. All products in the above categories were considered, and no limit was set on the minimum whole grain content for inclusion in the analysis.

From the total list of 226 foods containing grains (including 6 additional foods from non-grain food groups, 5 yogurts and 1 baby food), a sub-list of 76 potential whole grain products was extracted. After a further screening, 13 foods were excluded as they were found not to contain whole grain. Overall, 63 food items were found to contain whole grain, of which 29 individual food items were codified at brand level (fortified products or foods for special purpose). The remaining 34 foods which had a generic food code also had corresponding brand information recorded in the food consumption database.

Details regarding the whole grain content of food products with available brand information were obtained from the specific label of the product collected during the survey period (2005–2006 years), from the Mintel market research database [40] or from manufacturer’s websites. When brand-specific data were not available, the average whole grain content of similar products was used, or in case of mixed dishes, the recipe values were applied. In the case of whole meal bread purchased in bakeries, quantitative ingredient declarations (QUIDs) are not required by law and there is no clear regulation on the specific amount of whole wheat flour that should be used in the recipe in order to call the bread “whole meal”. Furthermore, it is not possible to know whether “whole wheat flour” or white flour with added bran was used in the recipe. For the current analysis, it was assumed that all whole meal bakery bread was made with whole wheat flour and the whole wheat flour content was estimated based on the average content of three traditional recipes, equating to 49 %.

In summary, the whole grain content of the 63 foods was obtained as follow: 44 % of the products from the specific label or Mintel database, 33 % from the average of similar products, 6 % from recipes and the remainder (17 %) were assigned an estimated value based on knowledge of common whole grain foods (e.g. brown rice). For each product, the amount of total whole grain per 100 g and by grain source (i.e. wheat, oats, rice, maize/corn, barley, rye, other) was recorded in a whole grains database. Whole grain foods were re-aggregated as follow: (1) Bread; (2) Pasta; (3) Rice; (4) Wheat, Other cereals & flours; (5) Ready to eat Breakfast cereals (RTEBC); (6) Sweet biscuits; (7) Savoury fine bakery products; (8) Cakes and sweet snacks; and (9) Other foods (e.g. yogurt with cereals). The quantities of whole grain consumed and associated nutrient intakes were calculated, at individual level, as per capita/day amount, by meal and by eating occasion (portion). Nutrient density expressed as amount of dietary fibre, cholesterol, vitamins and minerals per energy (amount/10 MJ) was also calculated.

### Statistical analysis

The mean of the 3 days was used to estimate the whole grain consumption for each subject. Mean, standard deviation, median and percentiles of distribution of whole grain intakes by socio-demographic and lifestyle factors were calculated for the total population and for consumers only. Whole grain intakes are reported separately for children (3–9.9 years old, no. 193), adolescents (10–17.9 years old, no. 247), adults (18–64.9 years old, no. 2313) and the older adults (≥65 years old, no. 517), but for subsequent analyses, children and adolescents were merged as were the adults and the older adults due to the small sample size. As there are no specific quantitative recommendations for whole grain intake in Italy, the adequacy of daily whole grain intakes was assessed based on US recommendation of three servings per day (or 48 g/day) [41].

Tertile analysis was also carried out in relation to whole grain intakes. Comparison of mean daily macro- and micro-nutrient intakes in non-consumers and across tertiles of mean daily whole grain intakes was made for the two age groups using the Wilcoxon test or the Kruskal–Wallis test as appropriate, since the daily intake data were not normally distributed. In addition, mean daily intakes of food groups (g/day) in non-consumers and across tertiles of whole grain intakes were analysed. The Dunn’s post hoc test was used to identify the significant pairwise differences across the groups of subjects (non-consumers and the three consumers’ groups defined according to tertiles of consumption). Multiple logistic regression analysis, backward stepwise method, was used to assess the relationship between socio-demographic and lifestyle characteristics and whole grain consumption (yes vs. no), and was performed for adults/older adults and for children/adolescents separately. Results are presented as crude and adjusted odds ratios (OR) with 95 % confidence intervals in order to evaluate the probability of being whole grains consumers.

The adequacy of mean daily nutrient intakes in adults was compared between non-consumers of whole grain and across tertiles of whole grain intake using the Probability of Adequate Nutrient Intake index (PANDiet) [42]. The PANDiet uses the probabilistic approach to estimate the adequacy of the nutrient intake of an individual; it takes into account the number of days surveyed, the mean nutrient intake and its intra-variability, the nutrient reference value and its variability; the PANDiet is based on the mean of two scores: the adequacy and the moderation. The reference values of nutrients for Italian population to calculate the PANDiet index are reported in the Online resource—Table A. As the PANDiet index is not validated for children and adolescents, nutrient adequacy was assessed by calculating for each nutrient the ratio of the daily individual intakes to standard recommended amounts [43] by subject’s gender and age category.

For all the analyses, two-sided p values lower than 0.05 were considered statistically significant for all the tests applied. The analyses were performed using the Statistical Analysis System computer software package (SAS package version 9.01; SAS Institute Inc., Cary, NC).

## Results

Table 1 shows daily intakes of whole grain in the total population and consumers only by age groups and gender. In the total sample, children and adolescents consumed an average of 2.0 and 2.2 g/day of whole grain, respectively, whereas adults and the older adults consumed 3.8 and 3.3 g/day, respectively. Overall, 24 % of the sample of children, adolescents and adults consumed whole grain products during the 3-day recording period, whereas only 18 % of older adults were consumers of whole grain. Among female consumers, mean daily intake of whole grain increased with age (*P* < 0.01) and ranged from 6.0 g/day in children to 19.1 g/day in older adults. Similarly, for male consumers, mean intakes in adults (18.7 g/day) and older adults (16.4 g/day) were significantly higher than in the children/adolescents sample (10.1 g/day) (*P* < 0.01). Significant gender differences were observed only in adults/older adults both in total population and consumers only (*P* < 0.0001 and <0.05), whereby intakes were higher in women than in men when considering the total population (4.3 vs 2.9 g/day), whereas in consumers only the intakes were 15.4 g/day in females and 18.3 g/day in males. However, among adults aged 18–64 years, there were twice as many whole grain consumers among women (30.5 %) than among men (15.7 %). In the total sample of adults and older adults, whole grain intakes were significantly higher in people living in North-Western (5.4 g/day) and Central (5.4 g/day) regions compared to those living in the South and Islands (2.0 g/day). Moreover, whole grain intakes were higher in adults with a high education level with respect to those with a low level (data not shown). For the total population of children and adolescents, significant differences by socio-demographic and lifestyle factors were not found.

**Table 1:** Intake of whole grain (g/day) by gender and age groups (total population and consumers only)—mean, median, standard deviation (SD) and 97.5th percentile (P97.5)

Gender	Total population (no. 3270)							Consumers only (no. 745)							
Age class (years)	*n*	Mean	Median	SD	P97.5	P*	P**	*n*	%	Mean	Median	SD	P97.5	P*	P**
*Total population*															
Males	1472	2.8	0.0	10.9	29.5		<.0.001	252	17.1	16.6	9.8	21.8	93.7		NS
Females	1798	4.0	0.0	12.3	34.6			493	27.4	14.6	8.5	19.9	76.8		
Total	3270	3.5	0.0	11.7	32.6			745	22.8	15.3	9.1	20.5	81.6		
*Children/adolescents*															
Males															
3–9.9 years	94	2.7	0.0	7.8	31.8	NS		25	26.6	10.1	3.5	12.6	44.2	NS	
10–17.9 years	108	2.4	0.0	7.1	18.0			26	24.1	10.0	7.0	11.7	49.7		
Females															
3–9.9 years	99	1.3	0.0	3.5	11.3	NS		21	21.2	6.0	3.6	5.5	25.0	NS	
10–17.9 years	139	2.1	0.0	6.4	16.7			32	23.0	9.2	5.6	10.8	58.9		
Males and females															
3–9.9 years	193	2.0	0.0	6.0	17.7	NS	NS	46	23.8	8.2	3.5	10.1	44.2	NS	NS
10–17.9 years	247	2.2	0.0	6.7	16.7			58	23.5	9.5	6.3	11.1	49.7		
*Adults/older adults*															
Males															
18–64.9 years	1068	2.9	0.0	11.7	29.5	NS		168	15.7	18.7	10.6	24.1	98.2	NS	
≥65 years	202	2.7	0.0	9.9	17.0			33	16.3	16.4	11.7	19.5	98.2		
Females															
18–64.9 years	1245	4.5	0.0	12.6	38.3	0.0001		380	30.5	14.8	9.0	19.3	61.1	NS	
≥65 years	315	3.6	0.0	14.2	34.4			60	19.0	19.1	9.9	27.7	121.1		
Males and females															
18–64.9 years	2313	3.8	0.0	12.2	34.4	0.0070	<0.001	548	23.7	16.0	9.5	20.9	81.0	NS	0.0382
≥65 years	517	3.3	0.0	12.7	32.6			93	18.0	18.1	10.0	25.0	98.2		

*SD* standard deviation, *NS* non-significant

* *P* value from Wilcoxon test for comparison across age groups

** *P* values from Wilcoxon test for comparison by gender, age groups pooled

Table 2 reports the results of logistic regression analysis carried out on adults/older adults to determine predictors of whole grain consumption. Females, younger adults (18–64 years), subjects living in North-Western and Central regions compared to the South and Islands and those on a diet were significantly more likely to be consumers of whole grain. In addition, those who rarely consumed fortified foods and who reported poor knowledge of diet–health relationship, and infrequent reading of food labels were significantly less likely to be consumers of whole grain. No predictors were significant for the subsample of children/adolescents (data not shown).

**Table 2:** Odds ratios (OR) and 95 % confidence intervals (CI) from logistic regression analysis showing the association of whole grain consumption (yes vs no) with different predictor variables

Adults/older adults	Crude OR (95 % CI)	Adjusted OR (95 % CI)^a^
*Gender*		
Females versus males	2.09 (1.73–2.52)	1.78 (1.44–2.21)*
*Age class*		
18–64.9 versus ≥65 years	1.42 (1.11–1.81)	1.42 (1.07–1.87)*
*Geographical area*		
North-West versus South and Islands	2.42 (1.92–3.04)	2.15 (1.68–2.76)*
North-East versus South and Islands	1.23 (0.97–1.66)	1.20 (0.89–1.61)
Centre versus South and Islands	1.82 (1.41–2.35)	1.54 (1.14–2.07)*
*Dieting*		
Yes versus no	1.87 (1.50–2.33)	1.49 (1.16–1.91)*
*Sedentary time*		
<4 versus ≥4 h/d	0.93 (0.76–1.14)	0.75 (0.60–0.95)*
*Use of fortified foods*		
Never/sometimes versus always	0.34 (0.18–0.65)	0.37 (0.19–0.75)*
Often versus always	0.65 (0.32–1.31)	0.54 (0.25–1.16)
*Knowledge of food*–*health relationship*		
Do not know/poor versus good	0.44 (0.33–0.58)	0.65 (0.47–0.89)*
Sufficient versus good	0.65 (0.54–0.79)	0.82 (0.66–1.01)
*Reading food labels*		
Never/rarely versus often/always	0.50 (0.41–0.60)	0.73 (0.59–0.91)*

* Wald Chi-square test for individual parameters are significantly different from zero (*P* value <0.05)

^a^Variables are mutually adjusted

**Fig. 1:**
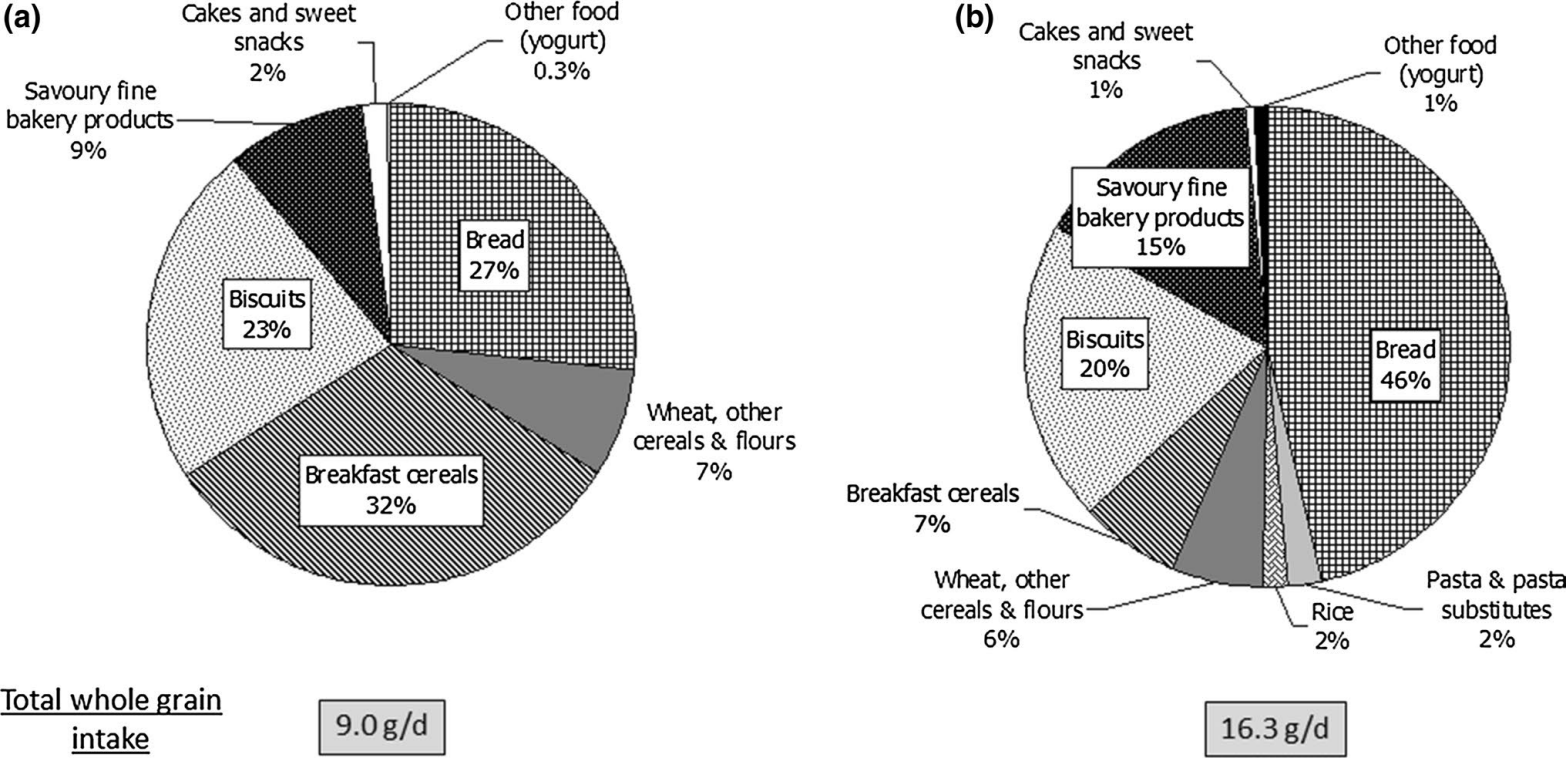
Contribution (%) of food categories to mean daily whole grain intakes for **a** Children/adolescents and **b** adults/older adults in whole grain consumers

Figure 1 shows the percent contribution from the different food groups to total whole grain intake in the consumers sample of children/adolescents and adults/older adults, respectively. In children/adolescents, the main food group contributor to whole grain intakes was RTEBCs (32 %), followed by bread and biscuits (27 and 23 %, respectively). In adults/older adults, bread was the main source, providing 46 % of whole grain intakes, while biscuits and savoury fine bakery products accounted for 20 and 15 %, respectively. In both age groups, wheat was the major type of grain contributing to whole grain intake, providing 65 and 80 % of total whole grain intakes, respectively, in children/adolescents and adults/older adults. Oats were the second highest contributor, providing 22 and 7 %, respectively.

The proportion of consumers achieving various levels of whole grain servings according to US whole grain recommendation is outlined in Table 3. The majority of children/adolescent consumers (63 %) had less than 1/2 servings per day (corresponding to <8 g/day), and about 17 % of them reached or exceeded 1 serving per day (≥16 g/day). Almost 69 % of adults/older adults consumers had less than 1 whole grain servings per day (<16 g/day), 19 % of them consumed between 2 and 3 servings per day, and only 5 % reached or exceeded the recommended 3 servings per day (48 g/day).

**Table 3:** Distribution of whole grain consumers by number of servings based on US whole grain recommendation [41]

	Number of servings														
	<1/2			≥1/2 to <1			≥1 to <2			≥2 to <3			≥3		
	<8 g/day			≥8 to <16 g/day			≥16 to <32 g/day			≥32 to <48 g/day			≥48 g/day		
	*n*	%	%*p*	*n*	%	%*p*	*n*	%	%*p*	*n*	%	%*p*	*n*	%	%*p*
Children/adolescents	65	62.5	14.8	21	20.2	4.8	13	12.5	3.0	3	2.9	0.7	2	1.9	0.5
3–9.9 years	29	63	15.0	10	21.7	5.2	5	10.9	2.6	2	4.4	1.0	0	0	0.0
10–17 years	36	62	18.7	11	19	5.7	8	13.8	4.1	1	1.7	0.5	2	3.5	1.0
*Adults/older adults*	287	44.8	10.1	156	24.3	5.5	47	7.3	1.7	120	18.7	4.2	31	4.8	1.1
18–64.9 years	248	45.3	10.7	131	23.9	5.7	39	7.1	1.7	104	19.0	4.5	26	4.7	1.1
≥65 years	39	41.9	7.5	25	26.9	4.8	8	8.6	1.6	16	17.2	3.1	5	5.4	1.0

%, percent on consumers only; %p, percent on total population (including non-consumers)

Mean daily intakes of energy, macro- and micro-nutrients in non-consumers versus consumers of whole grain across the tertiles of intake for children/adolescents and adults/older adults are shown in Tables 4 and 5, respectively. For adults/older adults, significant differences were observed for total energy, % total energy from total sugar, dietary fibre and several micro-nutrients. Percent total energy from total sugar in whole grain consumers (ranging 16.0–16.8 %) was significantly higher than in non-consumers (14.1 %) (*P* < 0.001), and energy-adjusted intakes of dietary fibre were significantly higher in whole grain consumers (ranging 22.2–26.1 g/day) than in non-consumers (20.9 g/day) (*P* < 0.001). Energy-adjusted mean intakes of most minerals (iron, calcium, potassium, phosphorus, zinc, magnesium) and vitamins (thiamine, riboflavin, vitamin C, vitamin B_6_ and vitamin A) were significantly higher as the daily intake of whole grain increased (*P* < 0.001). Although there was a trend for increasing dietary fibre and micro-nutrient intake with increasing whole grain intake in children/adolescents, significant differences only emerged for iron and magnesium (*P* < 0.05).

**Table 4:** Mean daily intakes of energy, macro-nutrients (as % of total energy intake), dietary fibre, vitamins and minerals (as 10 MJ) for non-consumer of whole grain and across tertiles of mean daily whole grain intakes in children/adolescents

	Non-consumers		Consumers						P*
			0.1–2.7 g/day		2.8–8.7 g/day		≥8.8 g/day		
	*n* = 336		*n* = 35		*n* = 35		*n* = 34		
	Mean	SD	Mean	SD	Mean	SD	Mean	SD	
Age (years)	10.8	4.3	10.5	4.0	11.4	3.4	11.7	4.1	NS
BMI *z* score^†^	0.50	1.79	0.84	2.09	0.32	1.55	0.26	1.48	NS
Energy									
(MJ/day)	8.8	2.7	9.2	2.3	9.8	2.7	9.3	2.4	NS
(kcal/day)	2093	639	2210	545	2348	656	2217	562	NS
% Energy from:									
Protein	15.7	2.2	15.8	2.3	15.7	2.0	15.8	1.9	NS
Fat	37.4	5.1	37.7	3.4	36.1	3.8	36.4	5.5	NS
Carbohydrates	46.8	5.9	46.3	4.4	48.0	4.6	47.6	5.8	NS
Total sugar	16.1	5.0	16.3	5.4	16.3	3.9	17.4	5.6	NS
Alcohol	0.1	0.3	0.0	0.0	0.1	0.2	0.0	0.2	NS
Dietary fibre (g/10 MJ)	17.6	4.5	18.2	3.8	18.7	4.3	20.5	7.7	NS
Iron (mg/10 MJ)	11.6	2.6	12.9	3.6	12.2	2.0	13.2	4.0	0.0134
Calcium (mg/10 MJ)	899	289	941	293	903	185	951	273	NS
Potassium (mg/10 MJ)	3083	655	3141	558	2941	555	3350	1041	NS
Phosphorus (mg/10 MJ)	1447	252	1515	213	1451	211	1494	229	NS
Zinc (mg/10 MJ)	12.5	2.4	12.5	2.6	12.3	2.0	13.1	2.3	NS
Magnesium (mg/10 MJ)	284	71	297	61	269^a^	47	331^b^	129	0.0240
Thiamine (mg/10 MJ)	1.13	0.26	1.26	0.40	1.15	0.21	1.28	0.38	NS
Riboflavin (mg/10 MJ)	1.69	0.46	1.80	0.38	1.71	0.35	1.86	0.56	NS
Vitamin C (mg/10 MJ)	134	81	149	86	142	69	162	173	NS
Vitamin B_6_ (mg/10 MJ)	2.2	0.5	2.2	0.7	2.2	0.5	2.5	0.8	NS
Vitamin A (µg/10 MJ)	877	1100	775	330	805	526	1041	1357	NS
Retinol (µg/10 MJ)	437	1023	347	135	338	92	508	1177	NS
β-Carotene (µg/10 MJ)	2637	2006	2570	1581	2802	3040	3201	2471	NS
Vitamin E (mg/10 MJ)	13.2	3.3	14.3	3.2	13.6	4.3	13.2	3.7	NS
Vitamin D (µg/10 MJ)	2.6	2.3	2.3	1.9	2.6	1.8	2.2	1.4	NS
Vitamin B_12_ (µg/10 MJ	7.1	5.8	9.0	7.3	6.8	4.1	7.5	5.1	NS

*SD* standard deviation, *NS* non-significant

* *P* values from the Kruskal–Wallis test, non-consumers versus tertiles of consumption

^†^The data on weight and height were self-reported. BMI *z* score based on age and gender specific was calculated using WHO AnthroPlus software version 1.0.4 (http://www.who.int/growthref/tools/en/)

^a,b^Mean values with unlike superscript letters were significantly different according to Dunn’s post hoc test for pairwise comparison, *P* < 0.05

**Table 5:** Mean daily intakes of energy, macro-nutrients (as % of total energy intake), dietary fibre, vitamins and minerals (as 10 MJ) for non-consumer of whole grain and across tertiles of mean daily whole grain intakes in adults/older adults

	Non-consumers		Consumers						P*
			0.1–5.7 g/day		5.8 to 14.9 g/day		≥15 g/day		
	*n* = 2189		*n* = 212		*n* = 217		*n* = 212		
	Mean	SD	Mean	SD	Mean	SD	Mean	SD	
Age (years)	49.0	17.4	45.8	15.8	47.5	16.1	48.7	15.5	NS
BMI (kg/m^2^)^†^	24.7^a^	3.9	24.0^b^	3.8	24.1	3.9	24.0^b^	3.7	0.0004
Energy (MJ)									
(MJ/d)	8.9^a^	2.6	9.0	2.8	8.3^b^	2.3	8.8	2.5	0.0086
(kcal/d)	2137^a^	619	2153	671	1984^b^	555	2104	601	0.0086
% Energy from:									
Protein	15.7	2.2	15.7	2.2	15.8	2.6	15.9	2.6	NS
Fat	35.9	5.5	36.5	5.3	36.3	5.6	36.0	5.2	NS
Carbohydrates	45.2	6.5	45.6	6.1	45.4	6.5	45.5	6.2	NS
Total sugar	14.1^a^	4.9	16.0^b^	5.0	16.8^b^	5.7	16.7^b^	5.0	<.0001
Alcohol	3.0^a^	4.2	2.1^b^	3.1	2.4	3.8	2.5	3.5	0.0030
Dietary fibre (g/10 MJ)	20.9^a^	6.7	22.2^b^	6.7	24.0^b^	8.3	26.1^c^	9.0	<.0001
Iron (mg/10 MJ)	12.7^a^	2.9	13.1^a,b^	3.2	13.9^c^	3.5	14.6^c,d^	3.3	<.0001
Calcium (mg/10 MJ)	852^a^	291	922^b^	288	953^b,c^	306	1016^c^	322	<.0001
Potassium (mg/10 MJ)	3426^a^	793	3585^b^	871	3837^b^	1118	3836^b^	1017	<.0001
Phosphorus (mg/10 MJ)	1418^a^	232	1441^a,b^	237	1468^b,c^	254	1562^c^	260	<.0001
Zinc (mg/10 MJ)	12.9^a^	2.6	13.1^a^	3.2	13.2^a^	3.2	14.0^b^	2.9	<.0001
Magnesium (mg/10 MJ)	309^a^	72	325^a,b^	79	341^b^	97	383^c^	105	<.0001
Thiamine (mg/10 MJ)	1.1^a^	0.3	1.2^b^	0.3	1.3^b,c^	0.4	1.3^c^	0.3	<.0001
Riboflavin (mg/10 MJ)	1.6^a^	0.4	1.8^b^	0.5	1.8^b^	0.6	1.9^b^	0.6	<.0001
Vitamin C (mg/10 MJ)	141^a^	90	154	99	159^b^	102	172^b^	116	<.0001
Vitamin B_6_ (mg/10 MJ)	2.2^a^	0.5	2.3	0.6	2.4^b^	0.7	2.4^b^	0.7	<.0001
Vitamin A (µg/10 MJ)	952^a^	1023	973	1037	1177^b^	1749	1169^b^	1384	<.0001
Retinol (µg/10 MJ)	368	909	357	930	431	1336	444	1233	NS
β-Carotene (µg/10 MJ)	3505^a^	2518	3693	2699	4476^b^	4324	4353^b^	3324	0.0003
Vitamin E (mg/10 MJ)	14.2^a^	3.7	14.7	4.4	15.3^b^	4.3	14.9	4.1	0.0007
Vitamin D (µg/10 MJ)	2.7	2.6	2.8	2.5	2.8	3.3	2.5	2.3	NS
Vitamin B_12_ (µg/10 MJ	6.8	5.9	6.6	5.8	7.0	7.6	6.6	5.6	NS

*SD* standard deviation, *NS* non-significant

* *P* values from the Kruskal–Wallis test, non-consumers versus tertiles of consumption

^†^The data on weight and height were self-reported. BMI data are missing for one female consumer in the 1st tertile

^a,b,c,d^Mean values with unlike superscript letters were significantly different according to Dunn’s post hoc test for pairwise comparison, *P* < 0.05

The mean of the PANDiet score of adult non-consumers of whole grain products was 59.23 and tended to increase with the increase in whole grain consumption, reaching 61.83 in the highest tertile of consumption (significant different at *P* < 0.0001). The adequacy score of non-consumers was significantly lower with respect to consumers, and so were the single items, except for total carbohydrates, total fat, polyunsaturated fatty acids, niacin, vitamin B_12_ and vitamin D; the moderation score was not significantly different. The only item that was significantly higher in consumers was the cholesterol (Online resource—Table B). In the children/adolescents sample, the adequacy of riboflavin, thiamine, vitamin B_12_ and iron intakes, calculated as the ratio of the daily individual intakes to standard gender and age recommended amounts [43], was significantly higher in whole grain consumers versus non-consumers (*P* < 0.05, online resource—Table C).

## Discussion

To the best of our knowledge, this study represents the first attempt to provide an evaluation of whole grain intakes in the Italian population. Our findings show a very low daily intake of whole grain in all age groups with only a quarter of the population reporting consumption of whole grain over the 3-day survey period. Furthermore, mean intakes among consumers ranged from only 6 g/day in female children to 19 g/day in female older adults and only 5 % of the adults/older adults sample achieved the US quantitative whole grain recommendation of 48 g/day. Wheat was the major source of whole grain provided mainly through consumption of bread and breakfast cereals.

The comparison of whole grain intakes between countries needs to be interpreted with caution as survey methods and sampling frames can differ [44] and the criteria for defining whole grain and a whole grain food may also vary from one country to the other [45]. Nonetheless, our results indicate that whole grain intakes are much below the reported intakes in other populations. Studies in the USA [29, 46], UK [21], Germany [25], Ireland [26, 27], Denmark, Norway and Sweden [24] reported a daily consumption of more than 13 g/day in children/adolescents (range 13–54 g/day) and 20 g/day in adults/older adults (range 20–51 g/day). Only in France [19] the whole grain intakes were comparable to those found in Italy (5 g/day in adults/older adults and 4 g/day in children/adolescents). Furthermore, the proportion of the Italian population reporting consumption of whole grain foods is well below the rate of consumers in other countries (ranging 32–90 %) [19, 20, 24–27, 29]. These low whole grain intakes are supported by a recent analysis of alkylresorcinol concentrations (a valid biomarker of whole grain wheat and rye intake) in the Italian cohort of the European Prospective Investigation into Cancer [47]. The main contributor to whole grain intakes was bread in adults/older adults, contributing about half of the intake, while breakfast cereals were the main source in children/adolescents (32 % of the total whole grain intake). Similar findings were reported in other European and US populations, suggesting that bread and breakfast cereals are well accepted whole grain foods among consumers. For example, bread accounted for approximately 30–80 % of total whole grain intakes in adults in France, Ireland, UK, Norway, Denmark and the USA [19–21, 24, 27, 29], while breakfast cereals accounted for 25–50 % of intakes in children/adolescents in Ireland, France and the USA [19, 26, 29]. Notably, biscuits made a substantial contribution to total whole grain intakes (~20 %) in Italian children and adults, while whole grain pasta did not appear in the diets of children and adolescents at all and contributed only 2 % to total whole grain intakes in adults.

The very low consumption of whole grain observed in the Italian population appears to be in paradox with the traditional Mediterranean diet which was first described in this region in the early 1960s and was characterized by a high intake of vegetables, legumes, fruits, olive oil, nuts, cereals (mostly unrefined), moderate intakes of milk and dairy products and fish and low intake of meat and meat products [48]. Over the last 50 years, the traditional Mediterranean diet has progressively disappeared in Italy. Food balance sheets for this period indicate that there has been a marked increase in the consumption of products of animal origin (meat and sausages, milk, cheese, animal fats) and a parallel decrease in the consumption of cereals, while the consumption of fruit and vegetables has increased slightly, as confirmed by the literature [49].

The reasons for such low intakes of whole grain in the Italian population are likely to be similar to those reported in other European countries and the USA which include a lack of knowledge on whole grain and its health benefits, difficulties in identifying foods made from whole grains, poor taste or texture perception of these products and the higher price [50, 51]. In line with these findings, we observed that those participants who practised more health conscious behaviours such as dieting, reading of food labels and use of fortified foods and who reported a good knowledge level on diet and health were more likely to be consumers of whole grain. Moreover, the subjects living in North-Western and Central regions compared to the South and Islands were significantly more likely to be consumers of whole grain, reflecting regional differences in the acceptance of whole grain foods. A study carried out as part of the HEALTHGRAIN project investigated consumer beliefs about whole grain products in four European countries and the impact of different types of health claims on the selection of grain products [50]. Interestingly, results showed that while Italian consumers generally rated whole grain foods positively in terms of their health benefits, they similarly rated refined grain foods and failed to identify the superior nutritional profile of whole grain above refined grain products. In addition, the use of health claims or whole grain labels did not positively influence Italian consumer’s willingness to buy whole grain products [52]. A further finding from this study indicates that perceived taste is another limiting factor to whole grain consumption in Italy as Italian consumers rated whole grain bread, pasta and biscuits as inferior in taste compared to their white flour alternatives [53, 54]. Hence, these findings present challenges for healthcare providers, educators and the food industry in promoting whole grain consumption in Italy, but they also give insights into potential strategies for addressing these barriers.

In both Denmark [55] and Singapore [56], successful national campaigns employing concurrent approaches which included increasing the availability of whole grain products and consumer awareness of their health benefits and the use of a specific logos to help consumers identify whole grain products resulted in significant increases in whole grain intakes in these populations. Similar targeted strategies may be effective in Italy with greater emphasis placed on increasing the awareness of the benefits of whole grain above refined grain and by increasing the availability of palatable whole grain alternatives to customarily consumed foods such as bread and pasta. As taste preferences are often established in childhood and can track into adulthood, exposing children to a range of whole grain foods from an early age is important. The school setting thus offers a suitable environment for early exposure to whole grain foods. In the USA since 2012, the National Schools breakfast and lunch programme must offer at least half of grains as whole grain in rich sources and this requirement increased to 100 % of grains offered for the 2014–2015 school year [57]. In some Italian municipalities, mainly in the Northern regions, the technical documents of school canteens recommend the use of whole wheat bread during meals served at lunch time, as suggested by the Italian Dietary Guidelines [35]. No quantitative advice is given, however, and the extent to which these guidelines are followed is unclear. Furthermore, these initiatives are carried out only at local level, a national directive on whole grain is not considered. However, an update of the Italian dietary guidelines is scheduled in the next year.

Another factor which may contribute to the low whole grain intakes in the Italian population is the lack of a quantitative recommendation for whole grain in the current Italian dietary guidelines [35]. Italians are advised to eat 5–10 portions of cereals (bread, rice, pasta, spelt, barley) according to energy requirement with only a suggestion to prefer whole grain products due to their naturally higher fibre content. A minimum target of whole grain servings is not specified. The Mediterranean Diet pyramid is slightly more descriptive recommending one or two servings of cereals per meal in the form of bread, pasta, rice, couscous and others and preferably whole grain versions, highlighting that some valuable nutrients (magnesium, phosphorus, etc.) and fibre can be lost during processing [36]. Very few national dietary guidelines define a specific quantity of whole grain: the USA recommends at least three 16 g servings per day [41] while the Danish recommend 75 g/day of whole grain (per 10 MJ energy intake) [58]. Estimated intakes in the Italian population fall substantially short of these recommendations with less than 5 % of the Italian consumers achieving the US target. Despite these low intakes, our findings showed that adults consuming ≥15 g/day of whole grain (equivalent to around one serving per day or more based on the US guidelines) had significantly higher intakes of dietary fibre and several minerals and vitamins compared to non-consumers of whole grain. This is consistent with findings from other population subgroups whose whole grain intake exceeds approximately 10 g/day [19, 21, 29]. Similar trends were also observed for children/adolescents but only reached significance for iron and magnesium, a finding which may be explained by the small sample size studied. Furthermore, the overall diet quality of adult whole grain consumers as measured by the PANDiet score tended to increase as whole grain intakes increased, and this appeared to be driven by more adequate intakes of dietary fibre, B vitamins (excluding niacin and B_12_), calcium, magnesium, zinc, phosphorous, potassium and iron (Online resource—Table B). The proportion of consumers achieving the recommended daily intake of dietary fibre (25 g/day) was twice as many (31 %) in the highest tertile of whole grain intake compared to non-consumers (14 %), suggesting that whole grain foods are a good vehicle for increasing fibre intakes in the Italian population which are currently sub-optimal in the majority of people [38].

These differences in nutritional intake can be accounted for, at least in part, the contribution of nutrients found in whole grain foods themselves. Whole grains contain higher amounts of fibre and several micro-nutrients including vitamin E, vitamin B_6_, folate, magnesium and zinc than refined grain [59]. For example, a comparison between the whole wheat bread versus the refined wheat bread in Italian Food composition database reveals a higher amounts of many nutrients: dietary fibre (6.5 vs 3.4 g/100 g), iron (25 vs 13.7 mg/100 g), zinc (1.6 vs 0.9 mg/100 g), magnesium (86 vs 15 mg/100 g), vitamin B_6_ (0.12 vs 0.07 mg/100 g), folate (72 vs 47 mcg/100 g). However, differences in nutrient intake may also result from a more careful approach to healthy food choices in general by consumers of whole grains. For example, adult/older adults consumers of whole grains consumed greater daily amounts of fruits and vegetables (477 g/day in consumers vs 431 g/day in non-consumers) and milk and milk products (218 vs 179 g/day), and less meat (102 g/day in consumers vs 113 g/day in non-consumers) and alcoholic beverages (83 g/day in consumers vs 113 g/day in non-consumers) than non-consumers of whole grain (Online resource—Table D). Finally, it should also be noted that there was a higher proportion of fortified foods consumers in the whole grain consumers group (adults and elderlies: 29 vs 9 %; children and adolescents 70 vs 25 %) which may have contributed to the higher micro-nutrient intakes observed in this group. For some of these nutrients (iron, riboflavin, thiamine, vitamin B_6_), this is mainly due to the higher consumption of fortified “Ready to eat Breakfast cereals”. The 28 % of “Ready to eat Breakfast cereals” consumers were present in the whole grain consumption group. Hence, the consumption of whole grain foods may contribute to and also act as a marker for a more healthful diet and healthier lifestyles.

There are several strengths and limitations of the current study that should be acknowledged. One of the main strengths was the nationally representative nature of the sample which covered all four main geographical areas and all classes of age. Furthermore, it was possible to estimate the intake of packaged whole grain products using the brands and labels of products consumed at the specific time of the dietary survey. However, the assessment of whole grain content of some products on the basis of current food labels and/or websites may be erroneous especially if products have undergone reformulation. The estimation of the whole grain content of wholemeal bread in particular may have been subject to error as most bread was purchased in bakeries and as such did not have corresponding QUIDs, unlike packaged bread. Moreover, we made an assumption that all bakery breads contained whole wheat flour rather than white flour with added bran so it is possible that some breads included in the current analysis were not whole grain as defined by the Health Grain Forum [23]. Thus, this could have resulted in an over-estimation of whole grain intakes. In addition, the effect of over- and under-reporting was not taken into consideration in the present analysis which may have resulted in an over- or under-estimation of whole grain intakes. A further limitation is the small number of children/adolescents in the studied sample which precluded analyses of statistical associations specifically in these age groups. Finally, the data presented were collected almost 10 years ago and may not reflect any recent changes dietary patterns including whole grain. A new national dietary survey is due to commence, however, in 2016/17, which should facilitate an estimate of trends in food consumption, included whole grains over the last decade.

In conclusion, whole grain was consumed in only a quarter of Italian children and adults, and among these, consumers intakes were substantially lower than quantitative whole grain recommendations. The main food sources of whole grain were breakfast cereals in younger people and bread in the adults, with wheat being the primary grain source. In spite of these low intakes, the positive association between whole grain consumption and dietary fibre and micro-nutrient intakes indicates that it could be an important vehicle for increasing intakes of these essential nutrients in the Italian diet. A greater understanding of the barriers to whole grain consumption affecting the Italian population is needed so that effective strategies to increase whole grain consumption can be devised.

## Electronic supplementary material

Below is the link to the electronic supplementary material.

Supplementary material 1 (PDF 297 kb)
