# Supplementary material for: Intakes of whole grain in an Italian sample of children, adolescents and adults
Source: Eur J Nutr. 2015 Nov 20;56(2):521–33. doi: 10.1007/s00394-015-1097-5 (PMC5334418; doi:10.1007/s00394-015-1097-5)
Supplement: Supplementary file 1 — Supplementary material 1 (PDF 297 kb) [file 394_2015_1097_MOESM1_ESM.pdf]

Article title: Intakes of whole grain in an Italian sample of children, adolescents and adults

Jounal name: European Journal of Nutrition

Author names: Stefania Sette, Laura D'Addezio, Raffaela Piccinelli, Sinead Hopkins, Cinzia Le Donne, Marika Ferrari, Lorenza Mistura, Aida Turrini

Corresponding author: Stefania Sette – crea - Consiglio per la ricerca in agricoltura e l'analisi dell'economia agraria – Centro di ricerca Alimenti e Nutrizione
Email: stefania.sette@entecra.it

Table A - Dietary reference values used for the PANDiet index

Items	Adequacy score		Moderation score	
	Reference value[1]	Variability[2]	Reference value[1]	Variability[2]
Total carbohydrate (% of energy[3])	45	0%	60	0%
Total Fat (% of energy[3])	20	0%	35	0%
SFA % (% of energy[3])	-	-	10	15%
Cholesterol (mg)		-	300	15%
Sodium (mg)	-	-	2000	15%
Protein (g/kg body weight)	0.71	13.8%		
PUFA (% of energy[3])	5	15%	Tolerable Upper Intake	
Dietary fibre (g)	25	10%		
Vitamin A (µg)	400 or 500	15%	3000	
Thiamine (mg)	0.9 or 1	15%		
Riboflavin (mg)	1.1 or 1.3	15%		
Niacin (mg)	14	15%	900	
Vitamin B_6 (mg)	1.1 or 1.4	15%	25	
Folate (µg)	1000	10%		
Vitamin B_{12} (µg)	2	10%		
Vitamin C (mg)	60 or 75	15%		
Vitamin D (µg)	10	15%		
Vitamin E (mg)	12 or 13	15%	300	
Calcium (mg)	800 or 1000	15%	2500/2000	
Magnesium (mg)	170	10%	250	
Zinc (mg)	7 or 9	15%	25	
Phosphorous (mg)	580	15%		
Potassium (mg)	3900	15%		
Iron (mg)	Based on tables by IoM[2]			

[1]Source: Dietary reference Intake of nutrients and energy for the Italian population [43]
[2] Source: Institute of Medicine Food and Nutrition Board 2001. Dietary Reference Intakes for Vitamin A, Vitamin K, Arsenic, Boron, Chromium, Copper, Iodine, Iron, Manganese, Molybdenum, Nickel, Silicon, Vanadium, and Zinc. Washington, DC: National Academy Press.
[3] as % of energy excluded alcohol
SFA = Saturated Fatty Acids
PUFA = Polyunsaturated Fatty Acids

Table B - PANDiet index for non-consumer of whole grain and across tertiles of mean daily whole grain intakes in adults/older adults

	Non-consumers n. 2189		Consumers 0.1 to 5.7 g/d n. 212		Consumers 5.8 to 14.9 g/d n. 217		Consumers ≥15 g/d n. 212		p*
	Mean	SD	Mean	SD	Mean	SD	Mean	SD	
PANDiet	59.23[b]	8.7	61.12[a]	9.03	60.23	9.09	61.83[a]	8.93	<.0001
Moderation	52.95	19.62	53.18	19.93	54.13	18.97	51.81	18.23	NS
Total Carbohydrates	0.98	0.11	1.00	0.03	0.96	0.14	0.98	0.12	NS
Total Fats	0.37	0.40	0.37	0.41	0.35	0.40	0.38	0.41	NS
SFA	0.34	0.37	0.33	0.37	0.31	0.37	0.31	0.36	NS
Cholesterol	0.60[b]	0.40	0.62	0.40	0.70[a]	0.38	0.68[a]	0.38	0.0002
Sodium	0.62	0.35	0.65	0.34	0.67	0.32	0.66	0.33	NS
Adequacy	65.51[6]	15.11	69.10 [c]	13.62	66.34 [b]	14.46	71.86	12.46	<.0001
Proteins	0.91[b]	0.19	0.92[a]	0.17	0.88	0.23	0.94	0.14	0.0092
Total Carbohydrates	0.62	0.41	0.64	0.40	0.60	0.41	0.62	0.41	NS
Total Fats	1.00	0.05	1.00	0.00	0.99	0.05	0.99	0.07	NS
PUFA	0.92	0.17	0.92	0.16	0.90	0.20	0.93	0.15	NS
Dietary fibre	0.19[b]	0.26	0.22[b]	0.27	0.21[b]	0.27	0.33[a]	0.32	<.0001
Vitamin A	0.74[b]	0.29	0.79	0.25	0.78	0.27	0.81[a]	0.27	0.0004
Thiamine	0.48[b]	0.33	0.56[a]	0.33	0.54	0.33	0.61[a]	0.31	<.0001
Riboflavin	0.63[b]	0.33	0.77[c]	0.26	0.69[b]	0.31	0.77[a]	0.27	<.0001
Niacin	0.73	0.29	0.73	0.30	0.70	0.30	0.78	0.24	NS
Vitamin B_6	0.86[b]	0.22	0.90	0.19	0.87	0.23	0.92[a]	0.16	0.0002
Folate	0.49[b]	0.37	0.56	0.35	0.48[b]	0.35	0.6[1a]	0.35	<.0001
Vitamin B_{12}	0.89	0.18	0.91	0.14	0.88	0.20	0.90	0.17	NS
Vitamin C	0.70[b]	0.33	0.78[a]	0.27	0.77	0.28	0.80[a]	0.28	<.0001
Vitamin D	0.03	0.11	0.04	0.12	0.03	0.11	0.03	0.09	NS
Vitamin E	0.46[b]	0.33	0.52[a]	0.33	0.46	0.33	0.51	0.33	0.0149
Calcium	0.37[b]	0.34	0.44[c]	0.34	0.41[b]	0.34	0.53[a]	0.36	<.0001
Magnesium	0.89[b]	0.22	0.91	0.20	0.90[b]	0.20	0.97[a]	0.10	<.0001
Zinc	0.80[b]	0.25	0.85[c]	0.23	0.79[b]	0.26	0.88[a]	0.18	<.0001
Phosphorous	0.97[b]	0.10	0.98	0.09	0.96[b]	0.10	0.99[a]	0.03	0.0005
Potassium	0.19[b]	0.24	0.22	0.27	0.21	0.25	0.26[a]	0.27	0.0002
Iron	0.88[a]	0.21	0.86[b,d]	0.22	0.87[b,d]	0.20	0.92[a]	0.14	0.0008

[a,b,c,d] Mean values with unlike superscript letters were significantly different according to Dunn's *post hoc* test for pairwise comparison, $p < 0.05$
* p values from the Kruskal-Wallis test, non-consumers vs tertiles of consumption
SD = Standard Deviation
NS = non significant
SFA = Saturated Fatty Acids
PUFA = Polyunsaturated Fatty Acids

Table C - Mean adequacy for non-consumer of whole grain and across tertiles of mean daily wholegrain intakes in children/adolescents.

	Non-consumers		Consumers						P*
			0.1 to 2.7 g/d		2.8 to 8.7 g/d		>=8.8 g/d		
	Mean	SD	Mean	SD	Mean	SD	Mean	SD	
	n. 336		n. 35		n. 35		n. 34		
Fats	1.25	0.17	1.26	0.11	1.20	0.13	1.21	0.18	NS
SFA	1.16	0.22	1.19	0.21	1.18	0.17	1.21	0.30	NS
Total sugars	1.08	0.33	1.09	0.36	1.09	0.26	1.16	0.37	NS
Dietary fibre	0.88	0.22	0.91	0.19	0.93	0.21	1.02	0.38	NS
Riboflavin	1.79	0.74	2.07	0.69	1.88	0.49	1.92	0.74	0.0461
Thiamine	1.46	0.56	1.73	0.64	1.55	0.42	1.64	0.64	0.0397
Vitamin C	2.40	1.74	2.80	1.90	2.77	1.66	2.65	2.12	NS
Vitamin B_{12}	4.11[a]	3.22	5.68[b]	5.19	4.11	2.24	4.28	2.55	0.0248
Vitamin A	2.09	2.57	1.96	0.90	2.09	1.46	2.41	3.01	NS
Calcium	0.81	0.31	0.93	0.36	0.89	0.24	0.89	0.31	NS
Iron	1.54 [a]	0.63	1.90 [b]	0.77	1.77	0.57	1.69	0.58	0.0037

[a,b,c] Mean values with unlike superscript letters were significantly different according to Dunn's *post hoc* test for pairwise comparison, p <0.05
*p values from the Kruskal-Wallis test, non-consumers vs tertiles of consumption
SD = Standard Deviation
NS = non significant
SFA = Saturated Fatty Acids

Table D1 - Daily food groups intakes (g/d) for non-consumer of whole grain and across tertiles of mean daily whole grain intakes in adults/older adults

	Non-consumers		Consumers						P*
			0.1 to 5.7 g/d		5.7 to 14.9 g/d		≥ 15 g/d		
	Mean	SD	Mean	SD	Mean	SD	Mean	SD	
	n. 2189		n. 212		n. 217		n. 212		
Cereals and cereal products	263.0[a]	103.9	258.8[ab]	116.7	232.5b[b]	98.5	251.0[ab]	92.6	0.0002
Legumes, fresh and processed	11.1	23.5	12.7	25.2	10.8	17.9	14.1	26.6	NS
Vegetables, fresh and processed	221.2	108.1	214.2	105.1	226.5	113.3	239.1	151.2	NS
Potatoes, tubers and products	51.4	55.3	55.0	59.3	45.0	53.8	48.7	55.1	NS
Fruit, fresh and processed	210.1[a]	158.8	237.3[b]	161.1	255.0[b]	162.5	258.9[b]	167.3	<.0001
Meat, meat products	113.2[a]	63.0	107.4[ab]	57.8	97.5[b]	59.1	101.8[b]	79.3	<.0001
Fish, seafood and their products	45.6	52.3	42.9	46.3	43.8	47.8	42.9	46.8	NS
Milk, milk products	178.6[a]	120.1	213.8[b]	112.8	204.9[b]	124.1	236.2[b]	142.3	<.0001
Oils & fats	41.7[a]	16.2	42.4[ab]	18.1	37.4[b]	15.6	40.1[ab]	16.9	0.002
Alcoholic beverages	113.3[a]	163.0	79.9[ab]	126.0	81.0[b]	121.2	89.3[ab]	132.6	0.002
Sweet products	31.8	32.5	35.0	30.6	35.4	34.7	34.7	31.4	NS
Meal substitutes	0.0	1.2	0.0	0.0	0.1	2.1	0.0	0.0	NS
Eggs	21.8	24.8	19.3	22.5	18.3	22.3	20.1	26.1	NS
Water and other non alcoholic beverages	824.7[a]	421.3	959.6[b]	461.8	939.9[b]	431.7	960.8[b]	498.2	<.0001
Miscellaneous	3.1	3.8	3.5	3.9	3.4	4.1	3.9	4.7	NS

[a,b,c] Mean values with unlike superscript letters were significantly different according to Dunn's *post hoc* test for pairwise comparison, p <0.05
*p values from the Kruskal-Wallis test, non-consumers vs tertiles of consumption
SD = Standard Deviation
NS = non significant

Table D2 - Daily food groups intakes (g/d) for non-consumer of whole grain and across tertiles of mean daily wholegrain intakes in children/adolescents

	Non-consumers		Consumers						P*
			0.1 to 2.7 g/d		2.8 to 8.7 g/d		≥8.8 g/d		
	Mean	SD	Mean	SD	Mean	SD	Mean	SD	
	n. 336		n.35		n.35		n.34		
Cereals and cereal products	264.6	111.1	285.4	96.0	268.9	77.2	313.3	103.6	NS
Legumes, fresh and processed	10.2	18.8	12.8	22.3	15.4	26.6	11.8	18.0	NS
Vegetables, fresh and processed	151.3	85.8	176.4	89.8	173.1	88.0	185.9	92.5	NS
Potatoes, tubers and products	55.9[ab]	54.7	50.1	55.0	39.9[a]	53.6	39.4[ab]	49.7	0.011
Fruit, fresh and processed	146.2	103.8	163.0	140.5	159.4	131.9	141.6	135.2	NS
Meat, meat products	111.7	54.5	118.3	59.8	103.9	69.3	126.6	53.1	0.043
Fish, seafood and their products	44.1	49.6	48.7	58.2	71.5	77.4	31.4	41.2	NS
Milk, milk products	234.5	122.2	267.0	119.8	275.8	126.1	267.2	103.1	NS
Oils & fats	37.4	15.6	39.3	16.1	41.0	14.6	40.0	17.1	NS
Alcoholic beverages	1.9	11.8	1.4	8.0	0.0	0.1	2.9	12.0	NS
Sweet products	38.4	37.5	44.4	41.8	42.3	38.8	42.4	41.6	NS
Meal substitutes	0.0	0.0	0.0	0.0	0.0	0.0	0.0	0.0	NS
Eggs	20.1[ab]	23.3	21.8	28.0	22.0[ab]	33.5	27.8[a]	24.8	0.011
Water and other non alcoholic beverages	757.7	373.9	804.4	384.9	798.5	366.0	857.2	495.5	NS
Miscellaneous	2.5[a]	3.3	3.4	3.4	3.0[ab]	3.3	4.1[b]	4.0	0.007

[a,b,c] Mean values with unlike superscript letters were significantly different according to Dunn's *post hoc* test for pairwise comparison, $p < 0.05$
*p values from the Kruskal-Wallis test, non-consumers vs tertiles of consumption
SD = Standard Deviation
NS = non significant
